# Extracorporeal shock wave therapy versus corticosteroid injection for chronic plantar fasciitis: A protocol of randomized controlled trial

**DOI:** 10.1097/MD.0000000000019920

**Published:** 2020-05-08

**Authors:** Jie Zhao, Wen Ming Luo, Tingting Li

**Affiliations:** a Department of Traumatic Orthopedics, Weifang People's Hospital, Weifang, Shandong, 261041; b Department of Ultrasound, Weifang Maternal and Child health Hospital, Weifang, Shandong, 261000, China.

**Keywords:** corticosteroid injection, extracorporeal shock wave therapy, plantar fascia, randomized controlled trial, study protocol

## Abstract

**Background:**

The outcomes of corticosteroid injection (CSI) and extracorporeal shock wave therapy (ESWT) as primary treatment of plantar fasciitis have been debated. This study was conducted to compare and evaluate the therapeutic effects of ultrasound-guided CSI versus medium frequency ESWT in the treatment of plantar fasciitis among Chinese population.

**Methods:**

This study was a single-center, randomized, and double-blinded trial. The study protocol was approved by local ethics committee board and subsequently registered in Research Registry. Eighty patients with unilateral plantar fasciitis were randomized to receive either ESWT (3 times once per week) (n = 40) or CSI treatment (a single 1-mL dose of betamethasone sodium plus 0.5 mL of prilocaine under ultrasound guidance by injection into the plantar fascia) (n = 40). The primary outcome measures were visual analog scale and Foot Function Index scores. Secondary outcome measures included the heel tenderness index score and plantar fascia thickness as obtained by ultrasound examination. All of the assessments were performed at baseline and 1, 3, and 6 months after treatment.

**Results:**

This is a randomized controlled trial evaluating the efficacy of CSI versus ESWT in the treatment of plantar fasciitis. This study has limited inclusion and exclusion criteria and a well-controlled intervention.

**Conclusions:**

The results of this trial will provide more evidence on which method can better treat plantar fasciitis.

**Trial registration:**

This study protocol was registered in Research Registry (researchregistry5428).

## 1 Introduction

The plantar fascia is the fibrous tissue that provides static support to the longitudinal arch on the bottom of the foot. Plantar fasciitis is a common cause of chronic plantar heel pain in the adult population and represents approximately 11% to 15% of all foot symptoms.^[1,2]^ Although its pathogenesis is not clear, possible increased risk factors include weak foot biomechanics, intrinsic muscle weakness, long periods of standing and walking, decreased elasticity of the plantar fascia, higher body mass index (BMI; calculated as the weight in kilograms divided by the square of the height in meters), and foot deformities such as pes planus.^[3,4]^ The pain is usually felt on the bottom of the heel and is most intense with the very first steps of the day or when the person starts to walk after a rest. The diagnosis is usually made on the basis of patient history and clinical findings. Physical examination may show local tenderness and swelling in the medial tubercle of the calcaneus. Ultrasonography is also used to visualize the plantar fascia and its insertion owing to its noninvasive and radiation-free nature, good tolerability, and relatively low cost.^[5]^ Ultrasonographic findings include blurred margins, decreased echogenicity, and thickening of the plantar fascia.^[1,6,7]^

Current treatments for plantar fasciitis are conservative and include rest, non-steroidal anti-inflammatory drugs (NSAIDs), stretching of the plantar fascia, physical therapy, foot padding, and orthotic devices, which can be used to suit patient needs.^[8]^ Other treatments for plantar fasciitis include local steroid injections, platelet-rich plasma, and intralesional botulinum toxin A.^[9]^ Corticosteroid injections are an effective and popular method to treat this condition. Nevertheless, serious side effects following corticosteroid injections, such as subsequent plantar fascia rupture, have been reported. Other treatments for plantar fasciitis, such as extracorporeal shock wave therapy (ESWT) and surgery, are recommended if patients do not respond to conservative treatments for at least 6 months.^[10]^ Shock waves in medicine are pulsed acoustic waves characterized by a short duration of time (<10 microseconds), very high pressure amplitudes, and relatively low tensile wave components (approximately 10% of the maximum pressure). Shock waves are generated outside the human body in water and transmitted widely over a large skin area onto the target region, where the acoustic energy is concentrated to a focal area 2 to 8 mm in diameter. It has been reported that ESWT is safe and efficacious in patients with chronic musculoskeletal disorders, such as tennis elbow, medial epicondylitis, tendinosis, and plantar fasciitis, who are resistant to conservative treatment.^[11,12]^

However, most of the published studies have compared focused ESWT at different intensities with corticosteroid injection,^[13,14]^ and we only identified one study that specifically compared rESWT with corticosteroid injection.^[15]^ The aim of this study was to compare the long-term clinical and ultrasonographic effects of rESWT and ultrasound-guided local corticosteroid injection treatment in patients with plantar fasciitis refractory to conservative treatment.

## 2 Materials and methods

### 2.1 Study design

**Figure 1:**
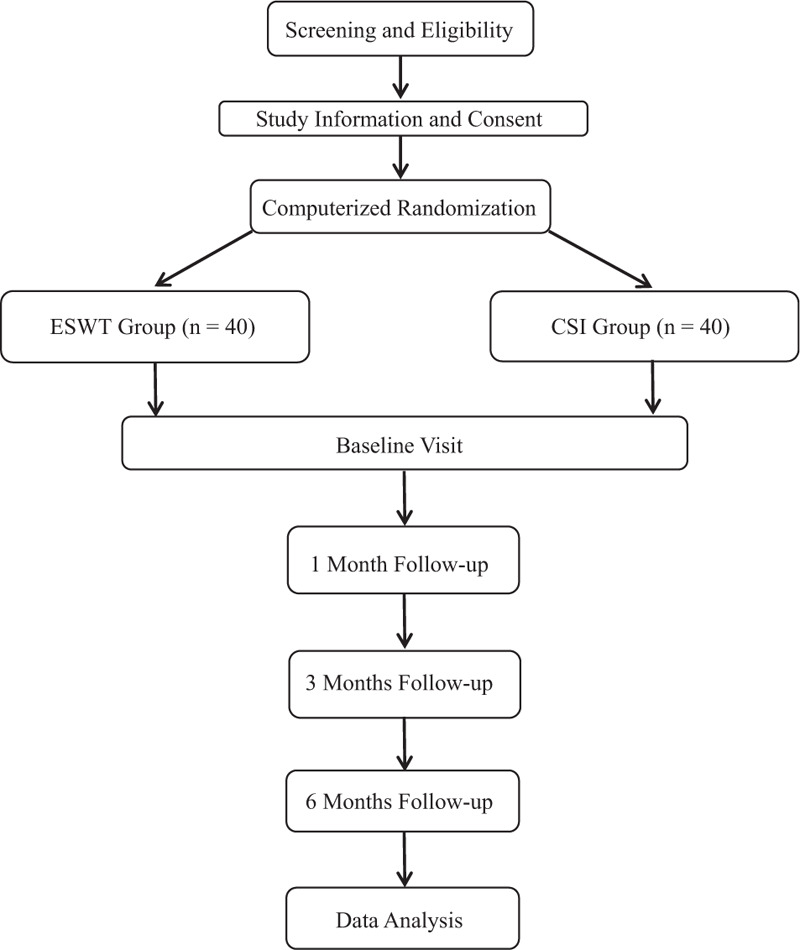
Flow diagram of the study.

This study was a prospective, 2-arm, parallel-group, open-label randomized controlled trial that is conducted at a single university hospital in China. We enrolled patients with chronic plantar fasciitis more than 2 months without injection history in the study. This study had been approved by the ethics committee of the Institutional Review Board (IRB) in our hospital (IRB No. 2031342). Informed consents were obtained from all patients in accordance with the Declaration of Helsinki. This systematic review protocol has been subsequently registered in Research Registry (researchregistry5428). The flowchart of this trial is shown in Figure 1.

### 2.2 Participants

We enrolled patients with chronic plantar fasciitis more than 2 months without injection history in the study. Patients with a cardiac pacemaker, previous surgery involving the foot, autoimmune or systemic inflammatory disorder, coagulation disorder or anticoagulant, calcaneal fracture, infections, pregnancy and peripheral neuropathy, or accepting other therapeutic modalities were excluded. The bilateral plantar fasciitis was excluded to produce a more homogenous group. The average symptoms duration before visiting was about 2 months. After 1-month conservative treatment failure, the patients maybe enroll in this study.

### 2.3 Randomization

After the baseline assessment and data collection, a computer-generated random number list was used to randomize patients into 2 equal groups: rESWT and local corticosteroid injection treatment. Randomization was performed using sequential sealed envelopes prepared by an independent physician before enrollment. The sealed envelopes contained a record of the treatment allocation. All investigators were blinded to the treatment allocation throughout the study.

### 2.4 Interventions

Patients were randomly assigned to 2 groups. The first group received rESWT and the other group was given corticosteroid injection. A Vibrolith Ortho (ELMED Ltd., Ankara, Turkey) ESWT device was used for rESWT application. Treatment was applied using a 15-mm head with 2000 shockwaves at each session at 10-Hz frequency with an energy flux density per shock of 0.16 mJ/mm^2^. Patients were in the prone position on the treatment bed with their feet hanging over the edge of the table. The applicator was placed on the plantar fascia insertion of the calcaneus. Each session was repeated 3 times per week by the same physiotherapist. Patients were not given local anesthetics. Except for transient skin redness at the administration site in 2 patients, no procedure-related problems occurred.

In the second group, before the corticosteroid injection the skin was prepped and draped. Then, 40 mg of methylprednisolone plus 1 mL of 1% lidocaine was injected under sterile conditions with a 22-gauge needle into the most painful tender point (usually in the medial plantar or inferior calcaneal area). A single injection was administered by an expert physiatrist without the guidance of sonography. Patients were recommended to have relative rest for 24 to 48 hours after injections and limit weight-bearing over the injected area. During this period, they were recommended to apply cold therapy 2 times a day for 10 minutes each time.

### 2.5 Outcome measurements

The primary outcome measures were visual analog scale (VAS) and Foot Function Index (FFI) scores. Secondary outcome measures included the heel tenderness index (HTI) score and plantar fascia thickness (PFT) as obtained by ultrasound examination. All of the assessments were completed by the same blinded investigator at baseline and 1, 3, and 6 months after treatment. Pain intensity is rated using a 100-mm horizontal VAS, for which 0 mm represent no pain and 100 mm represent extreme pain and compare between groups. The FFI was used to measure the impact of plantar fasciitis on foot function. This index consists of 23 items divided into 3 subcategories that address pain, disability, and limitation of daily life activities. Each item is rated by a VAS divided into ten equal parts. The values calculated for each subcategory indicate the degree of the respective functional disability, and the total score reflects the total foot dysfunction. The pain subcategory of the FFI questions both the pain that occurs with the very first steps in the morning and pain when standing or walking.

The HTI was used by the physician to assess heel pain, where 0 = no pain, 1 = painful, 2 = painful and winces, and 3 = painful, winces, and withdraws. All of the patients were also assessed using ultrasonography. Musculoskeletal ultrasound was performed by an experienced radiologist at baseline (before treatment) and 1, 3, and 6 months after treatment with an ultrasound device (Aplio 500; Toshiba Co Ltd., Tokyo, Japan) at B-mode using a 7.5- to 12-MHz superficial probe. Care was taken to maintain the blinding of the physician doing the ultrasound examination to the clinical data of patients. A linear probe was positioned longitudinally over the medial tubercle of the calcaneus. The PFT and its echogenicity were examined during ultrasonographic examination. The PFT was measured at the proximal point of insertion of the fascia into the calcaneal tubercle. A PFT of 4 mm or greater was considered evidence of fasciitis.

### 2.6 Sample size calculation

We estimate that with 32 participants in each group, the study will have more than 80% power to detect a clinically important difference between the groups in regard to the change in the pain score evaluated with the VAS. This is assuming a mean intergroup difference in score of 20 mm based on previous literature and a pooled standard deviation of 35 mm on the basis of preliminary data at an alpha level of 5%. Based on this estimation, a total of 80 patients are needed with an allowance for 10% drop-out.

### 2.7 Statistical analysis

The primary outcome of this study is compared between groups with a Student *t* test. For missing primary outcome data, VAS scores are replaced with the median scores for the other patients of the same treatment group at the same point in time. The comparisons between the study groups are performed with a chi-square test for categorical variables and a Student *t* test for continuous variables. All tests are 2-sided, and *P* < .05 is considered statistically significant.

## 3 Discussion

The plantar fasciitis is a self-limiting disorder; however, because of its prolonged course (mean period of 16 to 18 months), patients experience severe pain and disability affecting their quality of life. The diagnosis is based on patient history and clinical examination findings. Recently, ESWT has been recommended as an appropriate and effective method in the treatment of plantar fasciitis.^[4,5]^ ESWT modalities are capable of producing sonic waves with high amplitude within a short period and propagating them on a small surface. Theoretically, this energy could inhibit demyelinated plantar sensory nerves, reduce calcification, increase the proliferation of growth factors, and increase peripheral blood circulation, angiogenesis, and neovascularization in the degenerative tissue of the heel.^[8]^ Despite these theoretical views, the exact therapeutic effect of ESWT has not been substantiated by various clinical trials and a myriad of therapeutic treatment protocol regimens (e.g., the number of impulses, energy amount, shock wave frequency, focusing methods). Another important debate is the effectiveness of ESWT as a primary therapeutic regimen. The only controlled clinical trial evaluating radial ESWT versus a stretching technique demonstrated that the patients were not satisfied with the radial ESWT technique if it was applied as a primary treatment protocol.^[10–14]^

This trial has some limitations. First, the subjects may be exclusively Chinese. Therefore, the data from this clinical trial cannot be applied to other ethnic groups. Second, owing to the small sample size, the results of this study cannot be generalized. Despite these limitations, this trial is expected to provide more evidence on which method can better treat plantar fasciitis.

## Author contributions

Jie Zhao and Wen Ming Luo planned the study design and wrote the study protocol. Tingting Li reviewed the study protocol. Jie Zhao and Wen Ming Luo will recruit participants and collect data. Jie Zhao and Wen Ming Luo wrote the manuscript. All of the authors have read, commented on, and contributed to the submitted manuscript.

Tingting Li: 0000-0002-8606-9265.
